# Supplementary material for: Randomised Controlled Trial: Partial Hydrolysation of Casein Protein in Milk Decreases Gastrointestinal Symptoms in Subjects with Functional Gastrointestinal Disorders
Source: Nutrients. 2020 Jul 18;12(7):2140. doi: 10.3390/nu12072140 (PMC7400865; doi:10.3390/nu12072140)
Supplement: Supplementary file 1 [file nutrients-12-02140-s001.pdf]

Supplementary materials

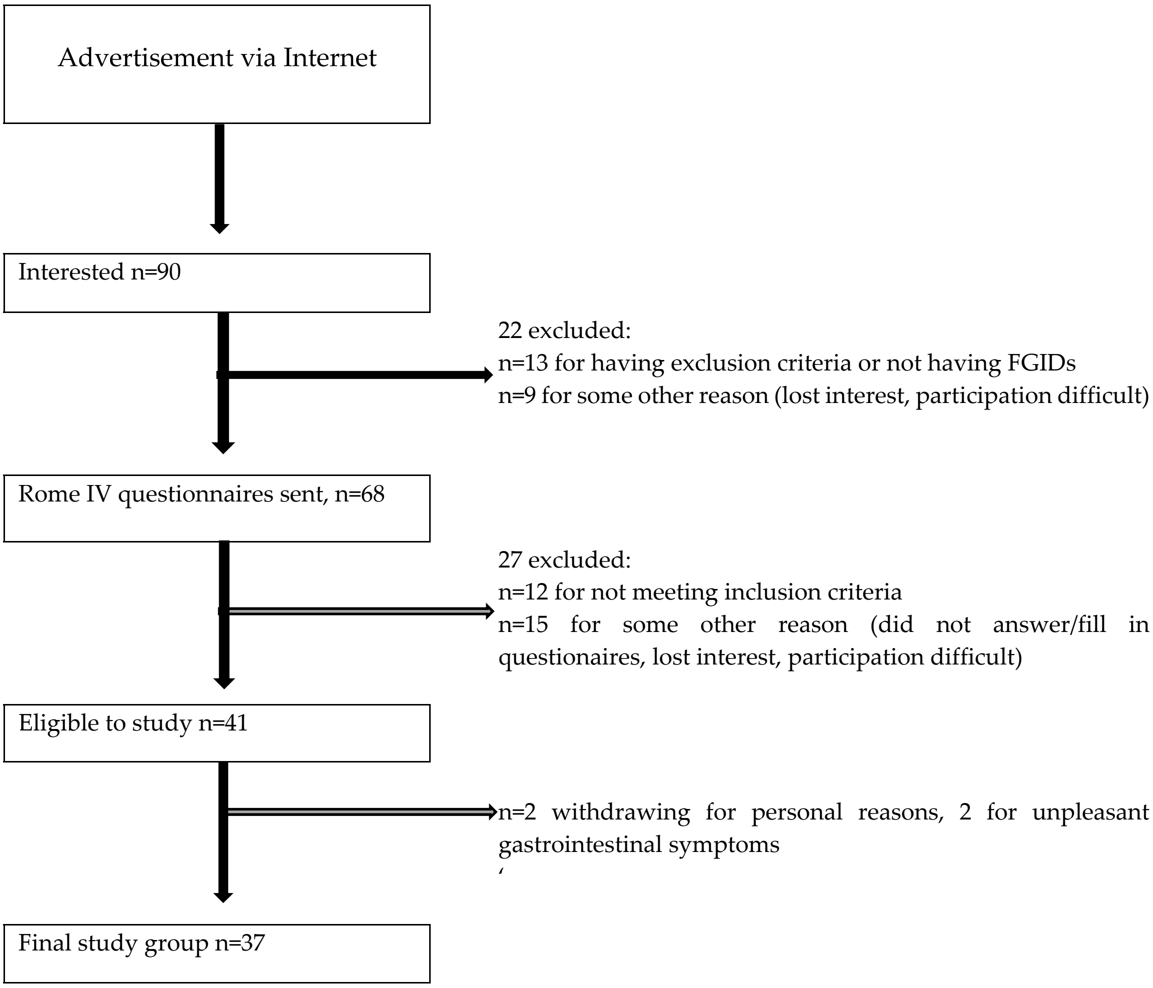

Figure S1. Flow chart.

Please rate the discomfort caused by the following gastrointestinal symptoms <u>today</u> on a scale from 1 to 5
(1=no discomfort, 5=severe discomfort)

	1	2	3	4	5
Abdominal pain	( )	( )	( )	( )	( )
Bloating	( )	( )	( )	( )	( )
Flatulence	( )	( )	( )	( )	( )
Rumbling	( )	( )	( )	( )	( )
Diarrhea	( )	( )	( )	( )	( )
Constipation	( )	( )	( )	( )	( )
Heartburn	( )	( )	( )	( )	( )
Rapid feeling of fullness	( )	( )	( )	( )	( )

Figure S2. Symptom diary.

Figure S3 (a-i).

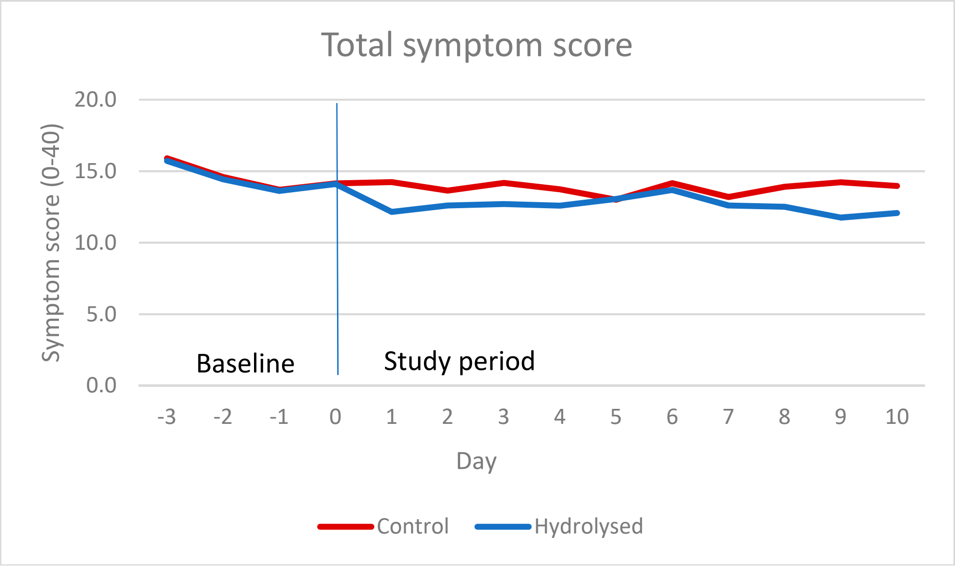

Figure S3.a. The development of total symptom score for gastrointestinal symptoms during the ten-day study periods

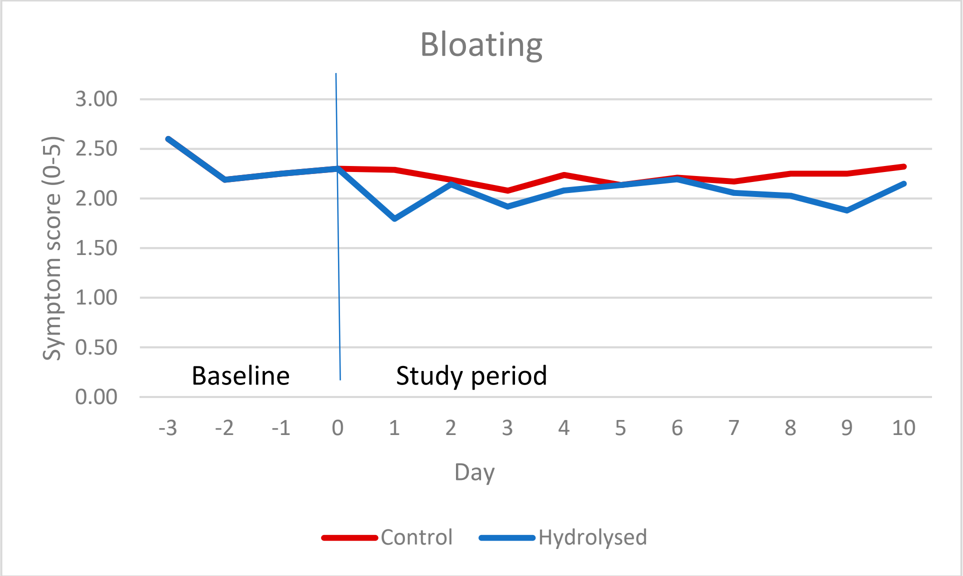

Figure S3.b. The development of bloating during the ten-day study periods

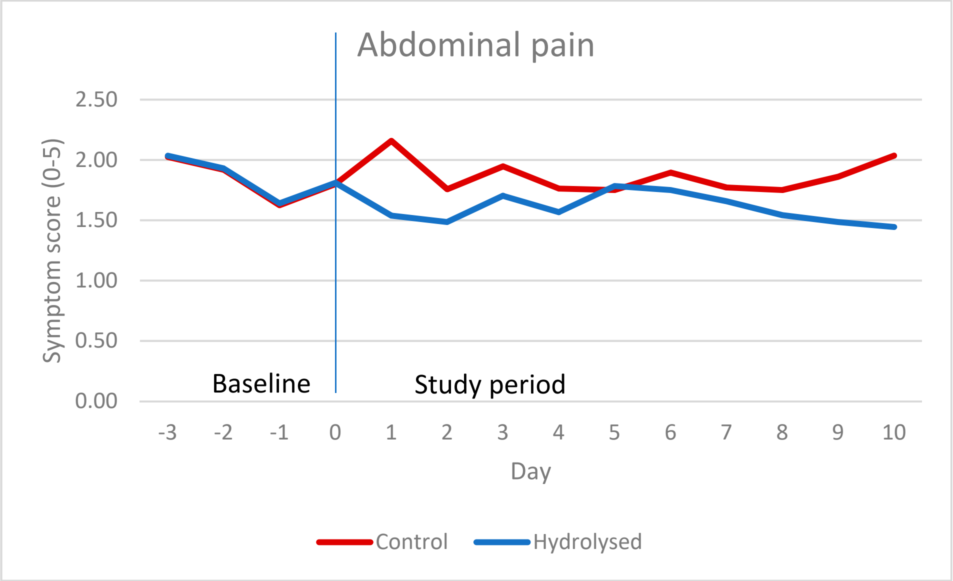

Figure S3.c. The development of abdominal pain during the ten-day study periods

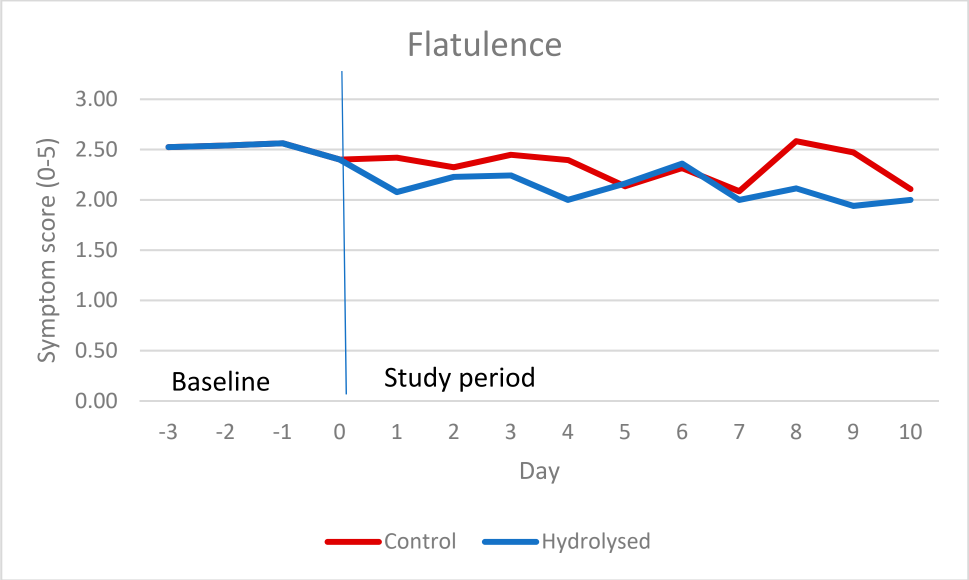

Figure S4.d. The development of flatulence during the ten-day study periods

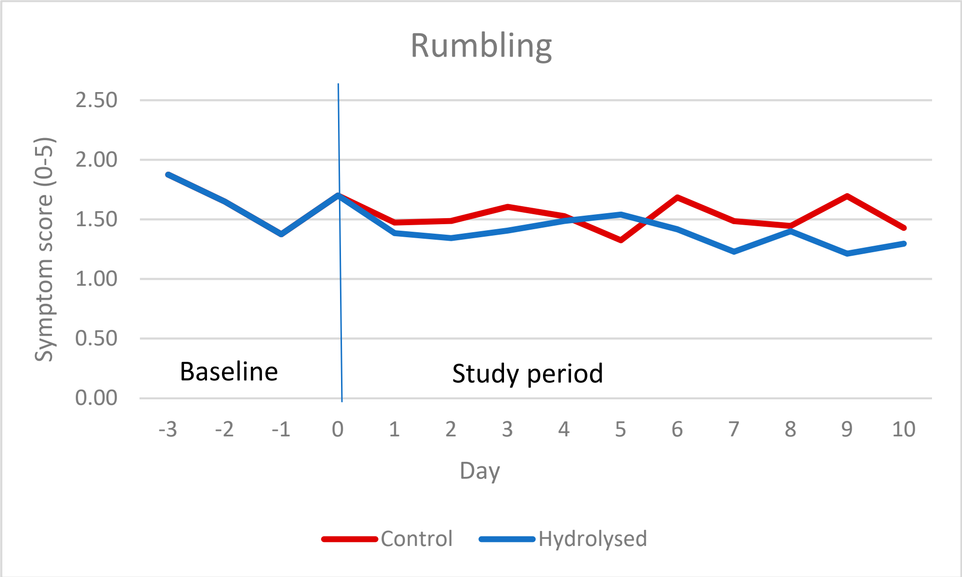

Figure S3.e. The development of rumbling during the ten-day study periods

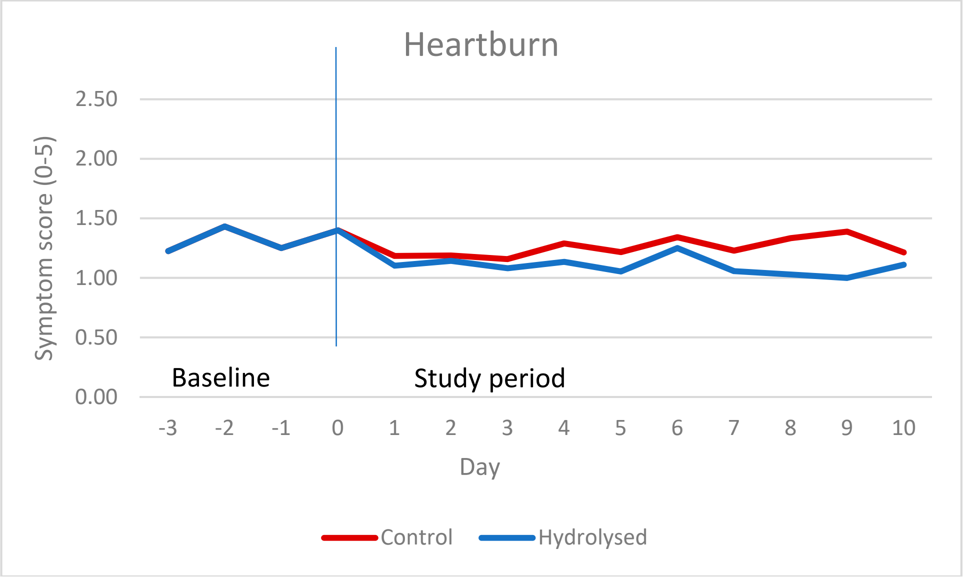

Figure S3.f. The development of heartburn during the ten-day study periods

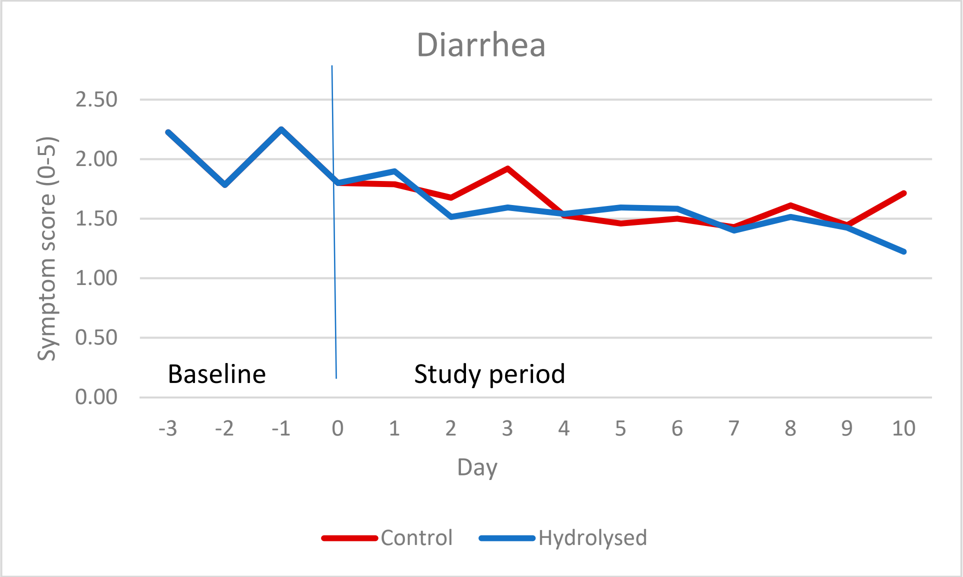

Figure S3.g. The development of diarrhea during the ten-day study periods

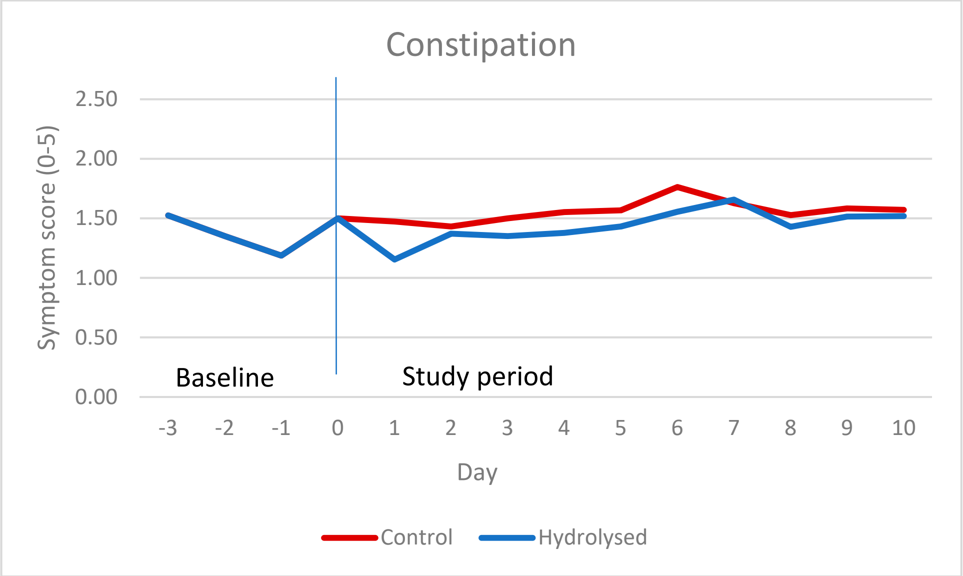

Figure S3.h. The development of constipation during the ten-day study periods

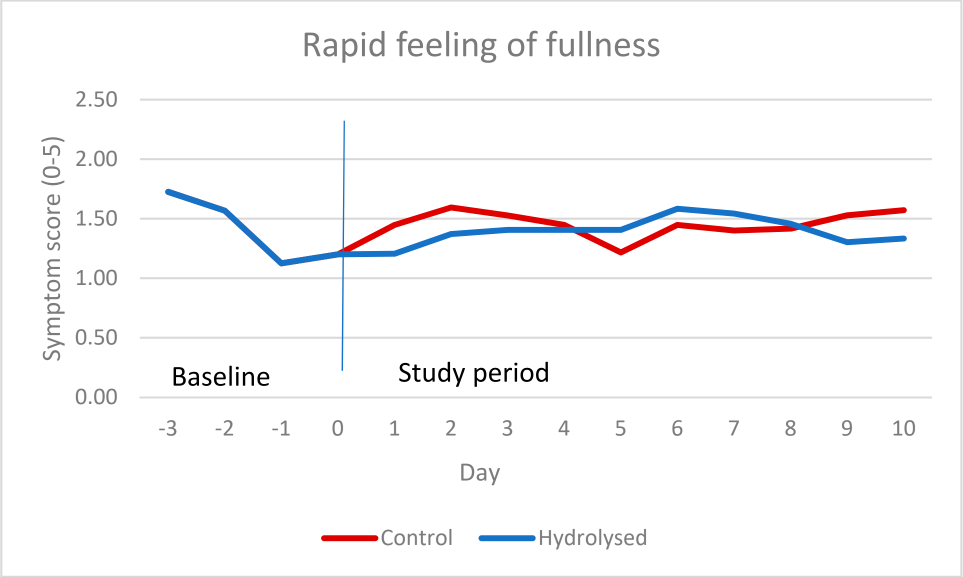

Figure S3.i. The development of a rapid feeling of fullness during the ten-day study periods
